# Oxidative Stress, Inflammation, and Activators of Mitochondrial Biogenesis: Tempol Targets in the Diaphragm Muscle of Exercise Trained-*mdx* Mice

**DOI:** 10.3389/fphys.2021.649793

**Published:** 2021-04-26

**Authors:** Heloina Nathalliê Mariano da Silva, Caroline Covatti, Guilherme Luiz da Rocha, Daniela Sayuri Mizobuti, Rafael Dias Mâncio, Túlio de Almeida Hermes, Larissa Akemi Kido, Valéria Helena Alves Cagnon, Elaine Cristina Leite Pereira, Elaine Minatel

**Affiliations:** ^1^Department of Structural and Functional Biology, Institute of Biology, State University of Campinas (UNICAMP), Campinas, Brazil; ^2^Faculty of Ceilândia, University of Brasília (UnB), Brasília, Brazil

**Keywords:** tempol, dystrophic muscles, exercise, oxidative stress, inflammatory process

## Abstract

The *mdx* mouse phenotype aggravated by chronic exercise on a treadmill makes this murine model more reliable for the study of muscular dystrophy. Thus, to better assess the Tempol effect on dystrophic pathways, the analyses in this study were performed in the blood samples and diaphragm muscle from treadmill trained adult (7–11-weeks old) *mdx* animals. The m*dx* mice were divided into three groups: *mdxSed*, sedentary controls (*n* = 28); *mdx*Ex, exercise-trained animals (*n* = 28); and *mdx*Ex+T, exercise-trained animals with the Tempol treatment (*n* = 28). The results demonstrated that the Tempol treatment promoted muscle strength gain, prevented muscle damage, reduced the inflammatory process, oxidative stress, and angiogenesis regulator, and up regulated the activators of mitochondrial biogenesis. The main new findings of this study are that Tempol reduced the NF-κB and increased the PGC1-α and PPARδ levels in the exercise-trained-*mdx* mice, which are probably related to the ability of this antioxidant to scavenge excessive ROS. These results reinforce the use of Tempol as a potential therapeutic strategy in DMD.

## Introduction

Duchenne muscular dystrophy (DMD) represents one of the most devastating types of muscular dystrophies, which affects one in 3,000–6,000 male children (Choi et al., 2016). DMD is an X-linked recessive genetic disease caused by a mutation in the dystrophin gene (Andrews and Wahl, 2018), for which to date, there is no cure.

Previous work showed that DMD is a multifactorial disease, whereby inflammation, mitochondrial dysfunction, altered angiogenesis, and oxidative stress are among the main promoters of the dystrophic features (Guiraud and Davies, 2017). Regarding oxidative stress, our research group has evaluated the effects of some antioxidant drugs on the dystrophic muscle of *mdx* mice, which was the experimental DMD model (Tonon et al., 2012; De Senzi et al., 2013; Mâncio et al., 2017a). Recently, we demonstrated that Tempol (4-hydroxy-2,2,6,6-tetramethylpiperidine-N-oxyl), a synthetic antioxidant that mimics the role of superoxide dismutase (SOD), improved the dystrophic phenotype (reducing myonecrosis and the inflammatory process) (Hermes et al., 2019) and contributed to the normalization of the redox homeostasis (Hermes et al., 2020) in young *mdx* mice during the acute dystrophic disease phase (about three weeks post-natal age). In addition, Burns et al. (2017) also reported that Tempol supplementation restores the diaphragm force and metabolic enzyme activities in adult *mdx* mice.

The present study further addresses the potential benefit of Tempol for dystrophy by evaluating the effects of this antioxidant in the diaphragm muscle in treadmill trained adult (7–11-weeks-old) *mdx* animals. Although the *mdx* mice lack dystrophin, similar to dystrophic DMD patients, these animals develop a milder form of dystrophy (Yucel et al., 2018). Thus, to get a more reliable animal model of the human dystrophy, the murine phenotype has been worsened by chronic exercise on a treadmill (Granchelli et al., 2000; De Luca et al., 2003). We carried out one of the most used protocols to worsen the murine phenotype based on the Treat-NMD Neuromuscular Network UK, 2018 for exercise in *mdx* mice. This consists of the *mdx* mice undergoing a 12 m/min exercise for 30 minutes twice a week. Under these conditions, the *mdx* muscle has often shown functional impairment indicated by the decline in grip strength, histopathological features of a worsening of the injury, and even a decrease of genes usually expressed in response to exercise (Camerino et al., 2014; Hyzewicz et al., 2015) enabling potential therapeutic interventions to be evaluated more rigorously throughout the *in vivo* treatment.

Consistent with the above actions of Tempol on the dystrophic muscle, we verified its potential pathways and effects on muscle damage, oxidative stress, inflammatory process, angiogenesis, and mitochondrial biogenesis in the blood samples and diaphragm muscle from adult exercised *mdx* mice.

## Materials and Methods

### Animals

Male m*dx* (C57BL/10-Dmdmdx/PasUnib) mice were used in all the procedures, which were previously approved by the Ethics Committee on the Use of Animals (CEUA) of the State University of Campinas (UNICAMP; #4847-1). The animals were housed and cared for following the guidelines of the Brazilian College for Animal Experimentation (COBEA). Mice chow and water were provided *ad libitum* and the animals were kept in a temperature-controlled room (25°C ± 0.5) with relative humidity (55% ± 1) and 12-h light/dark cycles.

### Experimental Design

Male m*dx* mice (seven weeks old; the age chosen was based on the previous exercise protocols) (Radley-Crabb et al., 2012; Hyzewicz et al., 2015) were randomly assigned into three groups: *mdxSed*, sedentary controls (*n* = 28); *mdx*Ex, exercise-trained animals (*n* = 28); and *mdx*Ex+T, exercise-trained animals with the Tempol treatment (*n* = 28).

**FIGURE 1:**
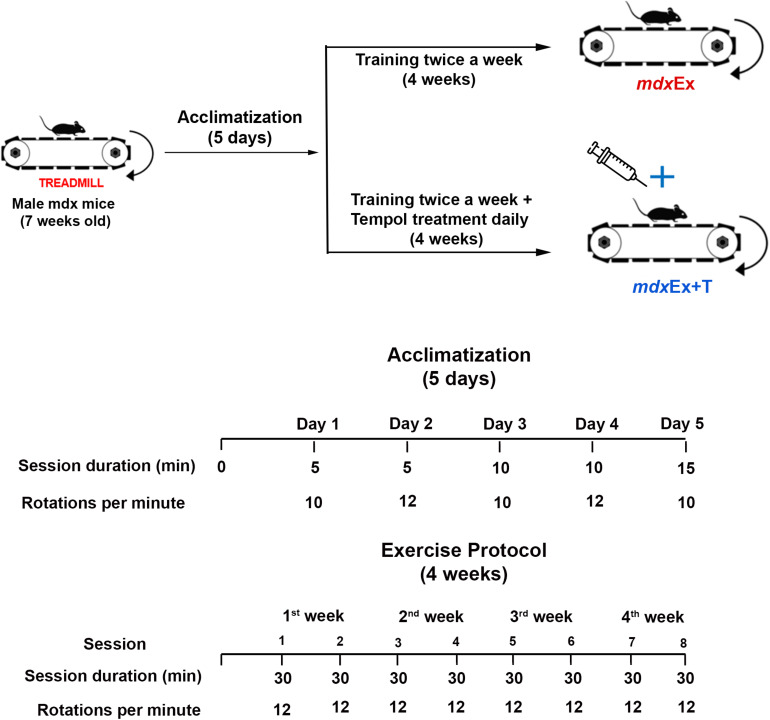
Exercise protocol and Tempol treatment.

The exercise protocol was based on the previous research (Radley-Crabb et al., 2012; Hyzewicz et al., 2015) using the TREAT-NMD protocols for exercise in *mdx* mice. After the five days of acclimatization, the *mdx* mice were trained for four weeks with progressively increasing loads (Figure 1). The protocol consisted of a treadmill exercise regimen of 30 min of treadmill running at a speed of 12 m/min twice a week for four weeks (keeping a constant interval of 2–3 days between each trial).

Tempol treatment began after the acclimatization period and continued daily until the day of euthanasia (Figure 1). The Tempol dose (100 mg/kg) dissolved in 100 μL of saline solution was administered intraperitoneally, according to our previous studies (Hermes et al., 2019; Hermes et al., 2020).

Twenty-four hours after the last training session, the animals were euthanized using a mixture of ketamine hydrochloride (130 mg/kg; Franco tar, Virbac, Fort Worth, TX, United States) and xylazine hydrochloride (6.8 mg/kg, 2% Virbaxil; Virbac). The blood samples were collected by cardiac puncture and the diaphragm (DIA) muscle was removed.

### Grip Strength Evaluation (*n* = 7 Per Group)

Forelimb muscle strength was evaluated with a grip strength meter (New Primer, São Paulo, Brazil), as previously described (Hermes et al., 2019). The measurements were obtained for each animal at the beginning and at the end of the experimental period and the absolute strength was normalized to body weight.

### Blood Samples for the Biochemical Assessment of Muscle Fiber Degeneration

For the creatine kinase (CK) assay, the blood samples (*n* = 7 per group) were micro-centrifuged in conical microtubes at 3,000 rpm for 10 min, and the supernatant (serum) was removed and used for analysis after incubation at room temperature for one to two hours to allow clotting. The CK assay was carried out using a commercially available kit (CK Cinetico Crystal, BioClin, Ireland) and a BioTek Spectrophotometer (BioTek Instruments Inc., Winooski, VT, United States). Values are reported as international units per liter.

### Histopathological Analysis (*n* = 7 Per Group)

#### Regenerated Muscle Fiber and Diameter Analyses

For the morphological analysis of the regenerated muscle fibers (central nucleated fibers) and normal fibers (peripheral nucleated fibers), cryosections of the DIA muscles were stained with hematoxylin/eosin. Slides were examined under a Nikon Eclipse TS100 microscope which is connected to a computer and a video camera (Nikon DS-Ri1). Non overlapping images of the entire cross-section were taken and tiled together using the NIS-elements AR Advances Research software (Nikon Instruments Inc., Melville, NY, United States) as described previously (Mâncio et al., 2017b). All the fibers of the cuts (normal and regenerated fibers) were counted to estimate the total population of the DIA muscle fibers. This allowed the percentage of normal and regenerated fibers of the studied animals to be obtained.

#### Minimum “Feret’s Diameter” Analyses

Feret’s diameter, the minimum distance of parallel tangents at opposing borders of the muscle fiber (Briguet et al., 2004), was analyzed by quantifying a total of a hundred muscle fiber diameter from 10 random fields at 20X magnification using the Image-Pro Express software (Media Cybernetic, Silver Spring, MD, United States). The results are shown as the mean ± SD.

#### Inflammatory Area

Inflammatory areas were characterized in hematoxylin/eosin stained serial cryosections (8 μm) of the DIA muscle based on the nucleus morphology and cell size and quantified as described previously (Hermes et al., 2019). Inflammatory cells are characterized by a poor cytoplasm and basophilic nuclear staining. Areas of inflammation and total muscle area were delimited. To limit these areas, the images were captured by light microscopy (Nikon Eclipse TS100) which is connected to a Nikon camcorder at 20X magnification and to a computer with a NIS-elements software AR Advances Researches. Areas of inflammation were expressed as a percentage of the total area. A blinded observer carried out the counts and measurements.

#### Macrophage Infiltration

F4/80 staining was used for macrophage infiltration quantification. Serial cryosections (8 μm) of the DIA muscle were fixed in acetone for 10 min, air-dried for 20 min, and washed with PBS. Sections were blocked for one hour at room temperature with 3% BSA in PBS. The histological slides were incubated overnight at 4°C with a primary antibody against F4/80, (1:200, monoclonal antibody; AbD Serotec, Raleigh, NC, United States). After washing with PBS, the slides were incubated with an anti-rat secondary antibody (1:250, Texas Red^®^ Anti-rat IgG; Vector Laboratories, Burlingame, CA, United States) for one hour at room temperature. After another washing with PBS, the muscle sections were mounted in 1,4-diazabicyclo[2.2.2]octane (DABCO; Sigma) mounting medium for fluorescence microscopy. The images of F4/80 staining were captured by light microscopy (Nikon Eclipse TS100) which is connected to a Nikon camcorder at 20X magnification and to a computer with a NIS-elements software AR Advances Researches. Macrophage infiltration areas (macrophages grouped in areas or dispersed by tissue) and total muscle area were delimited. Areas of macrophage infiltration were expressed as a percentage of the total area. A blinded observer carried out the counts and measurements.

#### Histopathological Oxidative Stress

For the quantification of the number of autofluorescent granules of lipofuscin, muscle samples were analyzed using serial cryosections unfixed (8 μm) of the DIA muscle. Quantification was performed in a fluorescent inverted microscope (Nikon, Eclipse TS100/TS100F) using the NIS-elements software AR Advances Researches in each cross-section (five cross-sectional areas per muscle).

Serial DIA cryosections (8 μm) were incubated with DHE (5 μl) to the quantification of the ROS levels. DHE staining presents a bright red emission in fluorescence microscopy. The intensity of reactive DHE by muscle area was quantified in a fluorescent inverted microscope (Nikon, Eclipse TS100) by measuring the pixels in a specific range (70 ± 255 wavelength). The equipment was adjusted to eliminate interference from background fluorescence.

### Western Blot Analysis (*n* = 7 Per Group)

Muscle samples were homogenized immediately after removal in 2 ml of lysis buffer (Tris–HCl, 100 mM, pH 7.5; EDTA, 10 mM, pH 8.0; sodium pyrophosphate, 10 mM; sodium fluoride, 0.1 mM; sodium orthovanadate, 10 mM; PMSF, 2 mM; and aprotinin, 10 μg/ml) using the Polytron PTA 20S homogenizer (model PT 10/35; Kinematica Ag) operated at maximum speed for 30 seconds. Muscle samples detritus were removed by centrifugation at 11,000 rpm for 20 min at 4°C and the cleared lysate was subjected to SDS-Page gel electrophoresis. The Bradford method was used to determine the total protein content. Total protein from the muscle sample lysate (30 μg) was stacked on 12% SDS-polyacrylamide gels.

The proteins were transferred from gels to nitrocellulose membranes by electrophoresis (Mini Trans-Blot^®^ electrophoretic transfer cell - Bio-Rad). A blocking buffer was used in all membranes for two hours at room temperature. Membranes were incubated with appropriate primary antibodies overnight at 4°C with gentle shaking. The following primary antibodies were used for Western blotting: 4-HNE (1:3,000, AHP1251, goat polyclonal, Bio-RAD, Hercules, CA, United States), anti-catalase (1:1,000, C0979, mouse monoclonal, Sigma-Aldrich, St. Louis, MO, United States), anti-SOD-2 (0,1:1,000, SAB 2501676, goat polyclonal, Sigma-Aldrich, St. Louis, MO, United States), anti-GSR (1:1,000, SAB2108147, rabbit, Sigma-Aldrich, St. Louis, MO, United States), anti-GPx1 (1:1,000, SAB2502102, goat polyclonal, Sigma-Aldrich, St. Louis, MO, United States), TNF-α (0,2:1,000, AAM19GA, rabbit anti-mouse polyclonal antibody; BIO-RAD, Hercules, CA, United States), NFkB p65 (1:1,000, AHP1342, rabbit polyclonal, BIO-RAD, Hercules, CA, United States), VEGF (1:200, sc53462, mouse monoclonal antibody; Santa Cruz Biotechnology), PGC-1α (1:1,000, ST1202, mouse monoclonal, Cambridge, United Kingdom), PPARδ (1:250, PA1-823A, rabbit polyclonal antibody, Invitrogen, CA, United States), Oxphos (6:1,000, ab110413, antibody cocktail, Abcam, Cambridge, United Kingdom), and β-actin (1:1,000, A1978, mouse monoclonal, Sigma-Aldrich, St. Louis, MO, United States).

Peroxidase-conjugated secondary antibodies from mice (1:2,500, 04-18-06, KPL, United States), goats (1:1,000, 14-13-06, KPL, United States), or rabbits (1:2,500, 04-15-06, KPL, United States) were used to incubate the membranes for two hours at room temperature. Membranes were washed 3 × 10 min with TBST after both incubations. Anti-β-Actin antibody was used as a control protein loading. All membranes were revealed using the Clarity Western ECL Substrate (Bio-Rad). Gene Tools from Syngene was used for bands intensity quantification.

### Glutathione (GSH) Content (*n* = 7 Per Group)

Total GSH content was determined by Ellman’s reaction using 5′5′-dithio-bis-2-nitrobenzoic acid (DTNB) as described by Anderson (1985). The intensity of the yellow color was read at 412 nm. The results were expressed as nmol per mg of protein.

### Gene Expression (mRNA) by Real-Time qPCR (*n* = 7 Per Group)

Total RNA from the *mdx* muscular tissue was extracted using the Trizol reagent according to the manufacturer’s instruction. RNA quantification was performed by spectrophotometry (260/280 nm). Synthesis of complementary DNA (cDNA) was performed from total RNA (2 μg) using the High-Capacity cDNA Kit (ThermoFisher). Gene expression was quantified by quantitative real-time PCR (ABI 7500 One Step quantitative PCR system–Applied Biosystems, CA, United States). The reactions were carried out in a mixture (20 μL) containing a sample of cDNA (2 μL), primers (300 nM), DEPC water, and SYBR Green PCR Master Mix (10 μL, Invitrogen). Gene expression levels were analyzed according to the ΔΔCT method (Livak and Schmittgen, 2001). qPCR analysis was performed in duplicate per sample on a plate. RPL39 was used as a reference gene. We used primers (Exxtend, Oligo Solutions, SP, Brazil) for Manganese Superoxide Dismutase (SOD 1*;* F: 5′-GCGGTGAACCAGTTGTGTTG-3′; R: 5′-CTGCACTGGTACAGCCTTGT′3′), Catalase (CAT*;* F: 5′- CTCGCAGAGACCTGATGTCC-3′; R: 5′-GACCCCGCGGTCA TGATATT-3′), Glutathione Peroxidase 1 (GPx1*;* F: 5′-TCCA GTATGTGTGCTGCTCAT-3′; R: 5′-TTCATCTCGGTGTAGTC CCG-3′), Glutathione-Disulfide Reductase (GSR, F: 5′-GG GGCTCACTGAAGACGAAG-3′; R: 5′-TCACAGCGTGATA CATCGGG-3′), and Ribosomal Protein L39 (RPL39, F: 5′-C AAAATCGCCCTATTCCTCA-3′; R: 5′-AGACCCAGCTT CGTTCTCCT-3′).

### Statistical Analysis

All data are expressed as mean ± standard deviation (SD). Statistical analysis for direct comparison between the means of groups was performed by ANOVA, followed by the Tukey test which was used for multiple statistical comparisons between groups. The normal distribution of the data was assessed by the Shapiro-Wilk normality test. A *p* ≤ 0.05 was considered statistically significant. We used the GraphPad Prims5 software package (GraphPad Software, CA, United States).

## Results

### Tempol Effects on Body Weight, Forelimb Muscle Strength, and Degeneration/Regeneration Muscular Process

There was no statistical difference in the weight of the animals during the experiment (Table 1). The *mdx*Ex group showed a significant reduction in muscle strength over the study period (Table 1). The *mdx*Ex+T group presented strength gain in the period, which was significantly different from the *mdx*Ex group (Table 1).

**TABLE 1:** Body weights, forelimb muscle strength, and CK levels.

	Body weight (g)		Body weight/períod	Force/Body weight (g/g)		Force gain/period	Creatine Kinase levels
	Start	End	(%)	Start	End	(%)	(U/L)
mdxSed	24.77 ± 1.43	28.16 ± 2.45	13.68	0.75 ± 0.05	0.77 ± 0.06	2.59	4,184 ± 3,442
*mdx*Ex	24.69 ± 1.24	27.67 ± 1.48	12.06	0.78 ± 0.17	0.55 ± 0.09***	−41.81	11,460 ± 1,149**
*mdx*Ex+T	24.24 ± 1.54	27.23 ± 2.18	12.33	0.72 ± 0.13	0.77 ± 0.11*^###^*	6.49	4,855 ± 1,756^##^

Body weight (g) was measured at the beginning (Start) and after four weeks (End) of the exercise and Tempol treatment. Forelimb muscle strength was assessed by taking measurements of force at time points Start and End, norm alized by body weight (g/g). Gain force/period: percentage of muscular force gain during the treatment period. Creatine kinase levels were measured at the end of the experiment. Experimental groups: sedentary *mdx* mice (*mdx*Sed), exercise trained mdx mice (*mdx*Ex), and exercise trained *mdx* mice treated with Tempol (*mdx*Ex+T). All values are shown as the mean ± standard deviation (SD) for 10 animals. **P ≤ 0.001 compared with the *mdx*Sed group, ***P ≤ 0.0001 compared with the *mdx*Sed group, ##P ≤ 0.001 compared with the *mdx*Ex group, ###*P* ≤ 0.0001 compared with the *mdx*Ex group (one-way .ANOVA with Tukey’s *post hoc* test).

The biochemical evaluation of the blood samples for CK levels showed a significant increase of this enzyme in the *mdx*Ex group (by 63.5%) compared to the *mdx*Sed group (Table 1). Concomitantly, there is a reduction of CK levels in the *mdx*Ex+T group (by 57.6%) compared to the *mdx*Ex group (Table 1).

**FIGURE 2:**
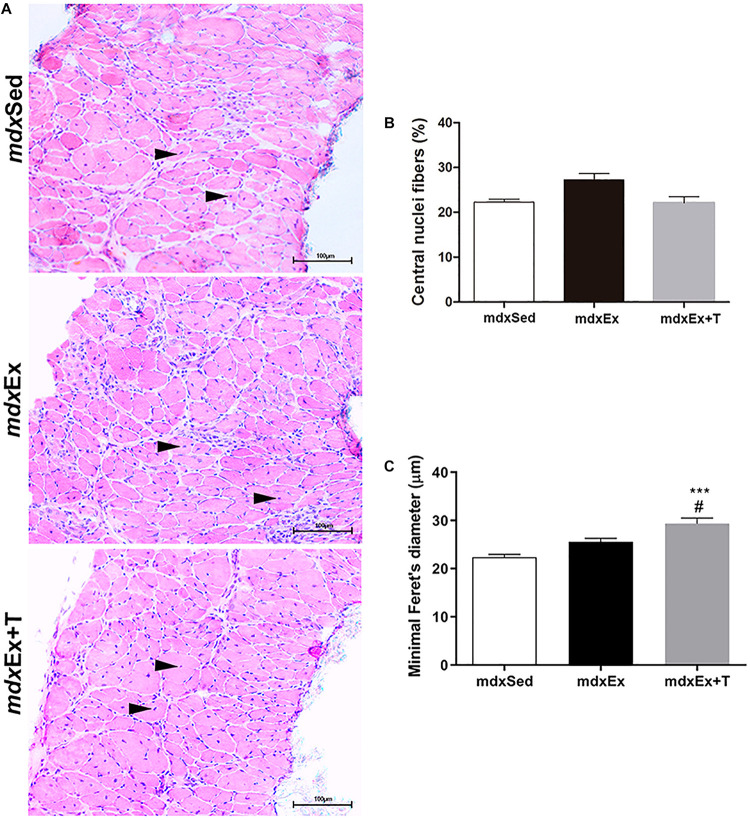
Tempol effects on the regenerative muscular process. **(A)** Diaphragm (DIA) cross-sections showing fibers with central nuclei (black arrowheads) in the sedentary *mdx* mice (*mdx*Sed), exercise trained *mdx* mice (*mdx*Ex), and exercise trained *mdx* mice treated with Tempol (*mdx*Ex+T). **(B)** Graphs show the nuclei central fibers (%) in the DIA muscles of the *mdx*Sed, *mdx*Ex, and *mdx*Ex+T groups. **(C)** Graphs show the cross-sectional size (Minimum “Feret’s diameter; μm) of the fibers with central nuclei in the DIA muscles of the *mdx*Sed, *mdx*Ex, and *mdx*Ex+T groups. All values expressed as mean ± standard deviation (SD). ***P ≤ 0.0001 compared with the *mdx*Sed group, ^#^*P* ≤ 0.05 compared with the *mdx*Ex group (one-way ANOVA with Tukey’s *post hoc* test).

The morphological evaluation of muscle fiber regeneration in the DIA muscles, based on the number of fibers with central nuclei, did not present a significant statistical difference between the analyzed groups (Figures 2A,B). Feret’s diameter analyses showed a significant increase in the diameter of the fibers with central nuclei in the *mdx*Ex+T group compared to the *mdx*Ex group (Figure 2C).

### Tempol Effects on the Inflammatory Process

The inflammatory process in the DIA muscle was evaluated by determining the inflammatory area, macrophage infiltration, NF-κB, and TNF-α content.

**FIGURE 3:**
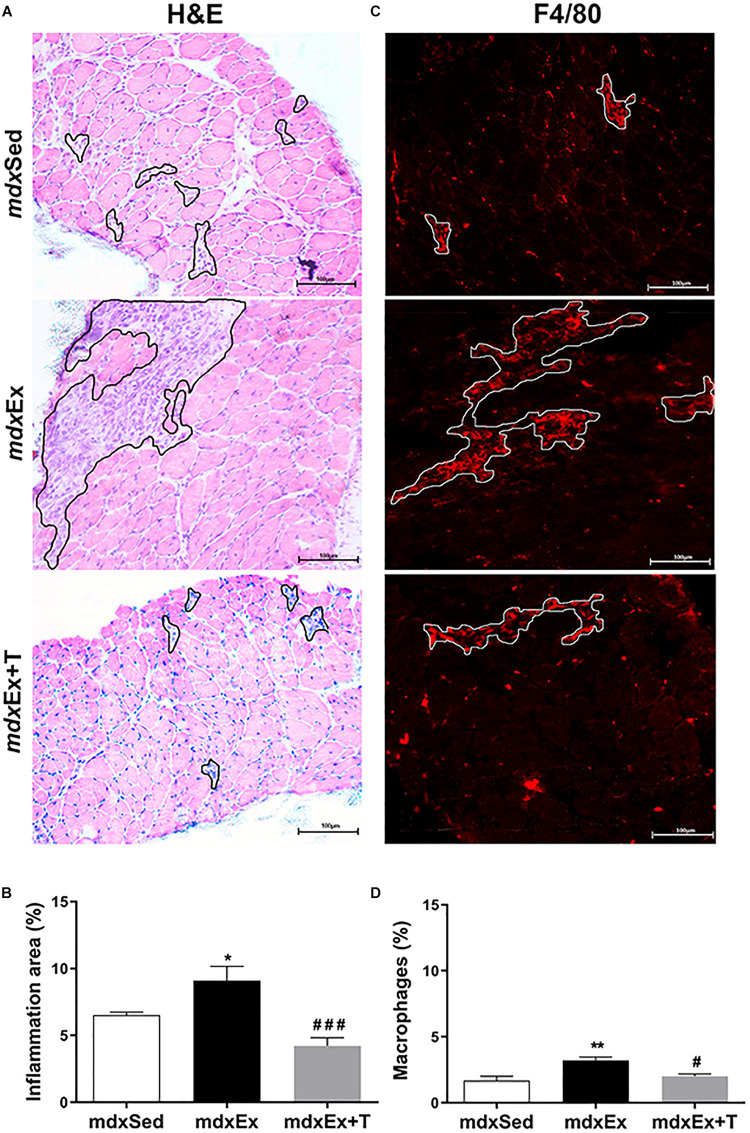
Tempol effects on the inflammatory response morphology. **(A)** The outline indicates the representative area of inflammation in the diaphragm (DIA) of the sedentary *mdx* mice *(mdx*Sed), exercise trained *mdx* mice (*mdx*Ex), and exercise trained *mdx* mice treated with Tempol (*mdx*Ex+T). **(B)** Graphs show the inflammatory area (%) in the DIA muscles of the *mdx*Sed, *mdx*Ex, and *mdx*Ex+T groups. **(C)** The outline indicates the representative area of macrophage infiltration determined by F4/80 immunohistochemistry. **(D)** Graphs show the macrophage infiltration area (%) in the DIA muscles of the *mdx*Sed, *mdx*Ex, and *mdx*Ex+T groups. All values expressed as mean ± standard deviation (SD). *P ≤ 0.05 compared with the *mdx*Sed group, **P ≤ 0.001 compared with the *mdx*Sed group, ^#^*P* ≤ 0.05 compared with the *mdx*Ex group, ^###^*P* ≤ 0.0001 compared with the *mdx*Ex group (one-way ANOVA with Tukey’s *post hoc* test).

The inflammatory area showed conspicuous regions containing inflammatory cells, which were densely packed among the muscle fibers (Figure 3A). The *mdx*Ex group showed an expressive inflammatory area (by 29.2%) compared to the *mdx*Sed group (Figure 2B). The diaphragm muscles of the *mdx*Ex+T group presented a significant reduction in the inflammatory area (by 54.7%) compared to the *mdx*Ex group (Figure 3B).

In terms of macrophage infiltration, the training-*mdx* mice showed a higher proportion (by 47.7%) compared to the *mdx*Sed group mice (Figures 3C,D). The *mdx*Ex+T group showed a significant reduction in macrophage infiltration (by 37.8%) compared to the *mdx*Ex group (Figures 3C,D).

**FIGURE 4:**
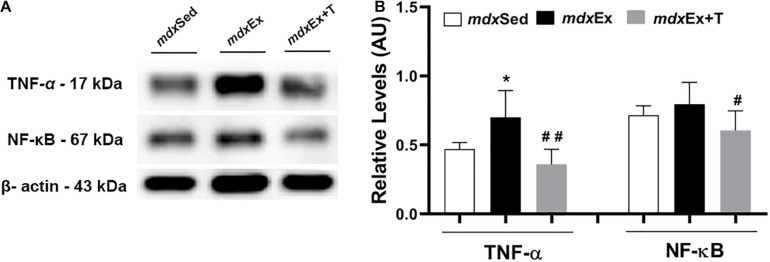
Tempol effects on the inflammatory markers. **(A)** Western blotting analysis of tumor necrosis factor alpha (TNF-α) and nuclear factor kappa B (NF-κB). The blots of the proteins (top row) and β-actin (loading control, bottom row) are shown. **(B)** The graphs show the protein levels in the crude extracts of diaphragm (DIA) from the sedentary *mdx* mice (*mdx*Sed), exercise trained *mdx* mice (*mdx*Ex), and exercise trained *mdx* mice treated with Tempol (*mdx*Ex+T). The intensities of each band were quantified and normalized to those of the corresponding control. Relative values are expressed as mean ± standard deviation (SD). *P ≤ 0.05 compared with the *mdx*Sed group,^#^*P* ≤ 0.05 compared with the *mdx*Ex group, ^##^*P* ≤ 0.001 compared with the *mdx*Ex group (one-way ANOVA with Tukey’s *post hoc* test).

Immunoblotting revealed a significant increase in the TNF-α levels in the *mdx*Ex group (by 33 and 32.8%, respectively) compared to the *mdx*Sed group (Figures 4A,B). The *mdx*Ex+T group presented a significant decrease in the TNF-α and NF-κB levels (by 49, 24, and 41%, respectively) compared to the *mdx*Ex group (Figures 4A,B).

### Tempol Effects on Oxidative Stress

**FIGURE 5:**
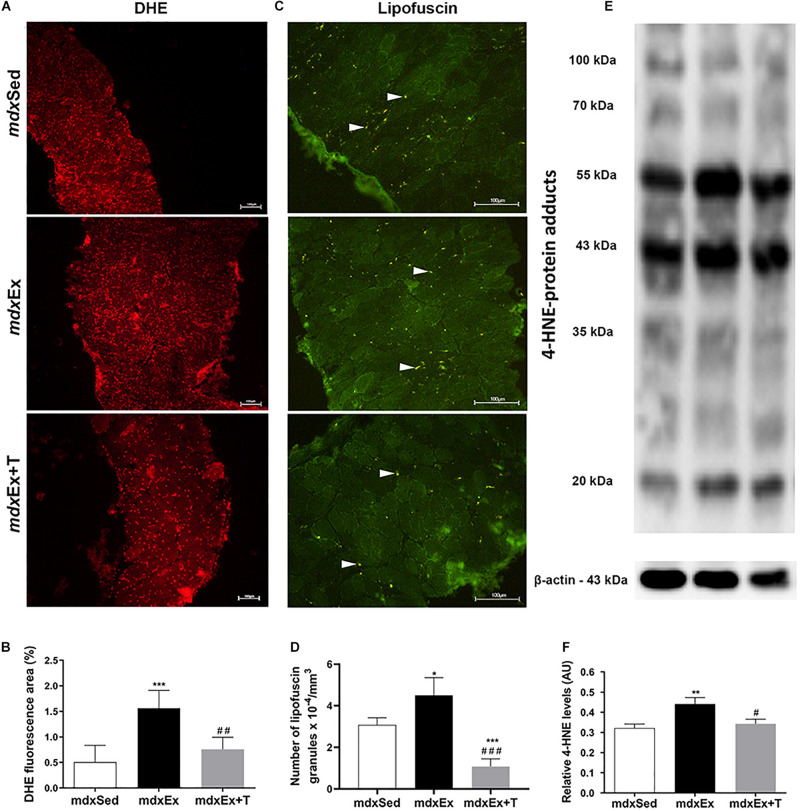
Tempol effects on the oxidative stress markers. **(A)** Diaphragm (DIA) cross-sections showing dihydroethidium (DHE) fluorescence in sedentary *mdx* mice (*mdx*Sed), exercise trained *mdx* mice (*mdx*Ex), and exercise trained *mdx* mice treated with Tempol (*mdx*Ex+T). **(B)** The graphs show the DHE staining area (%) in DIA muscles of the *mdx*Sed, *mdx*Ex, and *mdx*Ex+T groups. **(C)** DIA cross-sections showing autofluorescent lipofuscin granules (white arrowheads) in DIA muscles of the *mdx*Sed, *mdx*Ex, and *mdx*Ex+T groups. **(D)** The graphs show the number of lipofuscin granules x 10^– 4^/mm^3^ in DIA muscles of the *mdx*Sed, *mdx*Ex, and *mdx*Ex+T groups. **(E)** Western blotting analysis of 4-hydroxynonenal (4-HNE)-protein adducts. Bands corresponding to protein (top row) and β-actin (used as loading control) (bottom row) are shown. **(F)** The graphs show the protein levels in the crude extracts of DIA muscle from the *mdx*Sed, *mdx*Ex, and *mdx*Ex+T groups. All values expressed as mean ± standard deviation (SD). *P ≤ 0.05 compared with the *mdx*Sed group, **P ≤ 0.001 compared with the *mdx*Sed group, ***P ≤ 0.0001 compared with the *mdx*Sed group, ^#^*P* ≤ 0.05 compared with the *mdx*Ex group, ^##^*P* ≤ 0.001 compared with the *mdx*Ex group, ^###^*P* ≤ 0.0001 compared with the *mdx*Ex group (one-way ANOVA with Tukey’s *post hoc* test).

The DIA muscles of the *mdx*Ex group showed a significant increase in the oxidative stress markers, such as the DHE area, autofluorescent lipofuscin granules, and 4-HNE protein adduct levels (by 67.6, 29, and 27%, respectively) compared to the *mdx*Sed group (Figures 5A–F). The Tempol treated group presented a reduction of the oxidative stress markers by significantly decreasing the DHE area (51.5%), autofluorescent lipofuscin granules (69.4%), and 4-HNE protein adduct levels (22.4%) in the diaphragm muscle of the *mdx*Ex+T group (Figures 5A–F).

**FIGURE 6:**
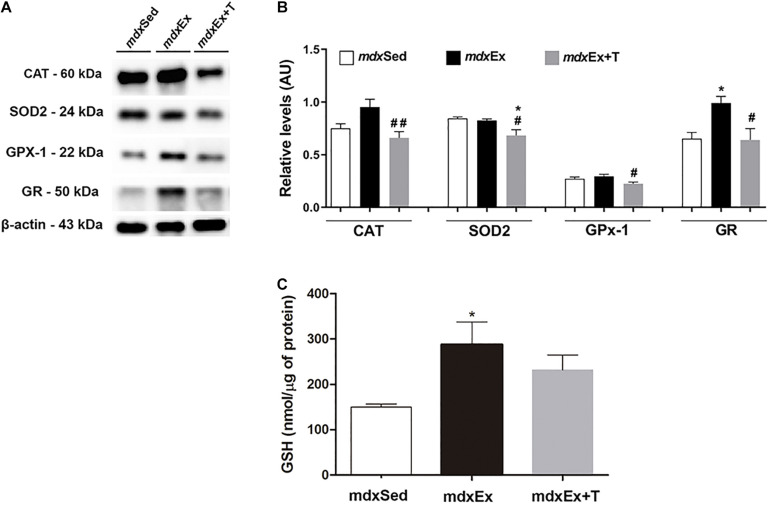
Tempol effects on the antioxidant system levels. **(A)** Western blotting analysis of catalase (CAT), manganese-superoxide dismutase (SOD2), glutathione peroxidase (GPx1), and glutathione reductase (GR) content in the diaphragm (DIA) muscle from the sedentary *mdx* mice (*mdx*Sed), exercise trained *mdx* mice (*mdx*Ex), and exercise trained *mdx* mice treated with Tempol (*mdx*Ex+T). The blots of the proteins (top row) and β-actin (loading control, bottom row) are shown. **(B)** The graphs show the protein levels in the crude extracts of DIA from the *mdx*Sed, *mdx*Ex, and *mdx*Ex+T groups. The intensities of each band were quantified and normalized to those of the corresponding control. **(C)** glutathione content of the DIA muscle from the *mdx*Sed, *mdx*Ex, and *mdx*Ex+T groups. Relative values are expressed as mean ± standard deviation (SD). *P ≤ 0.05 compared with the *mdx*Sed group,^#^*P* ≤ 0.05 compared with the *mdx*Ex group, ^##^*P* ≤ 0.001 compared with the *mdx*Ex group (one-way ANOVA with Tukey’s *post hoc* test).

The effect of the Tempol treatment on catalase, SOD2, GPx1, GR, and GSH immunoblots levels is shown in Figures 6A–C. The reduction of catalase, SOD2, and GPx1 levels (by 31.6, 15.6, and 24.9%, respectively) in the *mdx*Ex+T group was found to be significant when compared with the *mdx*Ex group (Figures 6A,B). The GR and GSH levels significantly increased (by 37.8 and 47.8%, respectively) in the *mdx*Ex group (Figures 6B,C) compared to the *mdx*Sed group. The *mdx*Ex+T group showed a significant decrease in the GR levels (by 35.2%) compared to the *mdx*Ex group (Figures 6A,B).

**FIGURE 7:**
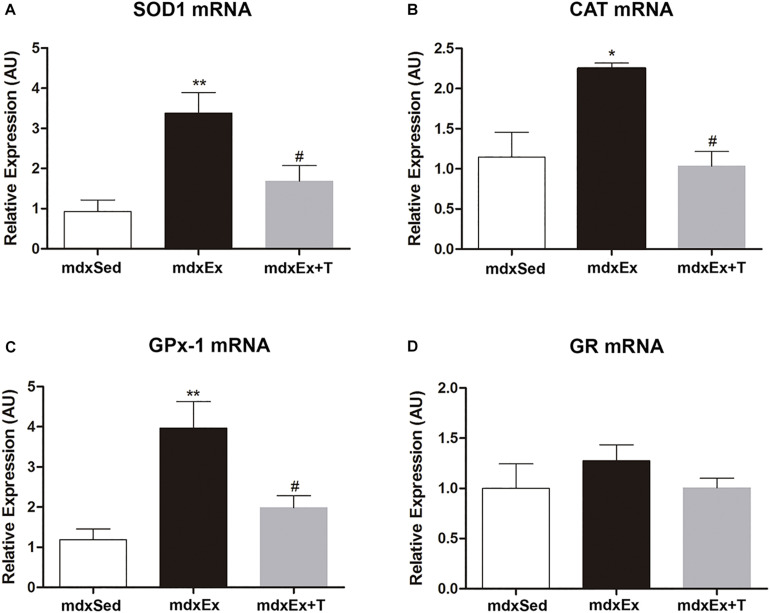
Tempol effects on the enzymatic antioxidant system expression. Gene expression of **(A)** manganese-superoxide dismutase (SOD), **(B)** catalase, **(C)** glutathione peroxidase (GPx), and **(D)** glutathione reductase (GR) measured by qRT-PCR in the diaphragm (DIA) muscle from the sedentary *mdx* mice (*mdx*Sed), exercise trained *mdx* mice (*mdx*Ex), and exercise trained *mdx* mice treated with Tempol (*mdx*Ex+T). All values expressed as mean ± standard deviation (SD). *P ≤ 0.05 compared with the *mdx*Sed group, **P ≤ 0.001 compared with the *mdx*Sed group, ^#^*P* ≤ 0.05 compared with the *mdx*Ex group (one-way ANOVA with Tukey’s *post hoc* test).

The effect of the Tempol treatment on the enzymatic antioxidant defense system gene expression is shown in Figures 7A–D. A significant increase in the gene expression of SOD1, CAT, and GPx was observed (by 72.5, 49.6%, and 70%, respectively) in the *mdx*Ex group when compared to the *mdx*Sed group (Figures 7A–C). In the *mdx*Ex+T group, there was a significant decrease in the SOD1, CAT, and GPx gene expression (by 50.1, 54%, and 49.9%, respectively) compared to the *mdx*Ex group (Figures 7A–C). There was no change in the GR gene expression between the experimental groups (Figure 7D).

### Tempol Effects on the VEGF Levels

**FIGURE 8:**
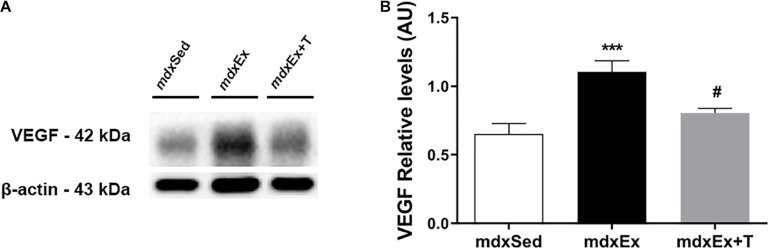
Tempol effects on the VEGF factor. **(A)** Western blotting analysis of the vascular endothelial growth factor (VEGF) content in the diaphragm (DIA) muscle from the sedentary *mdx* mice (*mdx*Sed), exercise trained *mdx* mice (*mdx*Ex), and exercise trained *mdx* mice treated with Tempol (*mdx*Ex+T). The blots of the proteins (top row) and β-actin (loading control, bottom row) are shown. **(B)** The graphs show the protein levels in the crude extracts of DIA from the *mdx*Sed, *mdx*Ex, and *mdx*Ex+T groups. The intensities of each band were quantified and normalized to those of the corresponding control. All values expressed as mean ± standard deviation (SD). ***P ≤ 0.0001 compared with the *mdx*Sed group, ^#^*P* ≤ 0.05 compared with the *mdx*Ex group (one-way ANOVA with Tukey’s *post hoc* test).

The DIA muscles of the *mdx*Ex group showed a significant increase in the VEGF levels (by 14.1%) compared to the *mdx*Sed group (Figure 8). A decrease of the VEGF levels (by 27.1%) were observed in the *mdx*Ex+T group compared to the *mdx*Ex group (Figure 8).

### Tempol Effects on the Activators of Mitochondrial Biogenesis and Oxidative Phosphorylation

**FIGURE 9:**
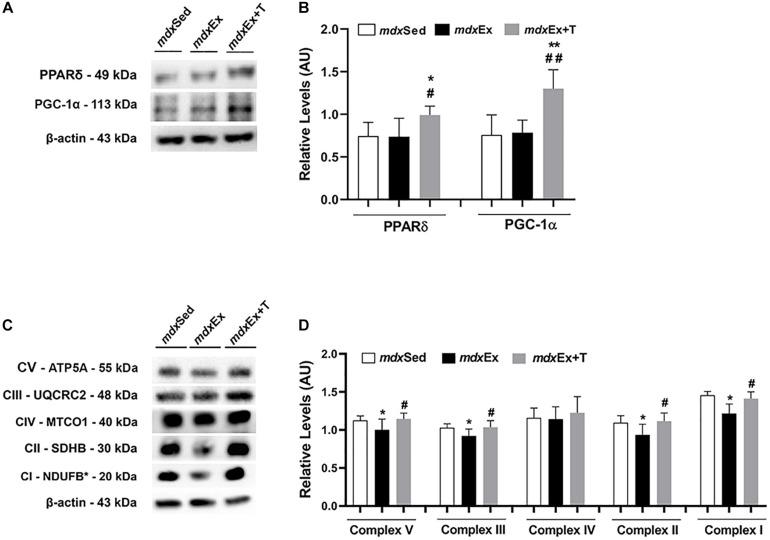
Tempol effects on the activators of mitochondrial biogenesis and oxidative phosphorylation. **(A)** Western blotting analysis of peroxisome proliferator-activated receptor δ (PPARδ) and peroxisome proliferator-activated receptor-γ coactivator-1α (PGC-1α) in the diaphragm (DIA) muscle from the sedentary *mdx* mice (*mdx*Sed), exercise trained *mdx* mice (*mdx*Ex), and exercise trained *mdx* mice treated with Tempol (*mdx*Ex+T). **(B)** The graphs show the PPARδ and PGC-1α levels in the crude extracts of DIA from the *mdx*Sed, *mdx*Ex, and *mdx*Ex+T groups. **(C)** Western blotting analysis of the OXPHOS complexes (complex V, III, IV, II, and I) in the DIA muscle from the *mdx*Sed, *mdx*Ex, and *mdx*Ex+T groups. **(D)** The graphs show the OXPHOS protein in the crude extracts of DIA from the *mdx*Sed, *mdx*Ex, and *mdx*Ex+T groups. The blots of the proteins (top row) and β-actin (loading control, bottom row) are shown. The intensities of each band were quantified and normalized to those of the corresponding control. All values expressed as mean ± standard deviation (SD). *P ≤ 0.05 compared with the *mdx*Sed group. **P ≤ 0.001 compared with the *mdx*Sed group. ^#^*P* ≤ 0.05 compared with the *mdx*Ex group. ^##^*P* ≤ 0.001 compared with the *mdx*Ex group (one-way ANOVA with Tukey’s *post hoc* test).

An increase in the PPARδ and PGC-1α levels was observed in the *mdx*Ex+T group compared to the *mdx*Sed (by 29.1 and 42.3%, respectively) and *mdx*Ex groups (by 25.8 and 40.0%, respectively) (Figures 9A,B).

The DIA muscles of the *mdx*Ex group showed a significant decrease in the OXPHOS levels (complex V, III, II, and I) compared to the *mdx*Sed group (Figures 9C,D). An increase in the OXPHOS levels (complex V, III, II, and I) was observed in the *mdx*Ex+T group compared to the *mdx*Ex group (by 12.6, 11.01, 16.1, and 13.9%, respectively) (Figures 9C,D).

## Discussion

M*dx* mice are the most widely used model to study DMD and test potential therapies, but their phenotype is milder than that of the dystrophic patient, thus, limiting the range of histopathological parameters and molecular changes that can be measured in pre-clinical drug tests (Grounds et al., 2008). In agreement with previous studies (Camerino et al., 2014; for review Hyzewicz et al., 2015), the present study found that exercise training substantially affects the dystrophic features of adult *mdx* mice by making them more closely resemble DMD.

Although muscle damage was found due to the high CK levels, the number of regenerated muscle fibers were not altered after the exercise. It has been demonstrated in literature that there is no absolute correlation between the extent of dystropathology in an individual *mdx* mouse and CK activity (Grounds et al., 2008). The increase in the CK serum levels indicates that the exercise was sufficient to render the muscular membrane as leaky and allow the release of CK into the circulation (Kobayashi et al., 2012). CK serological levels have long been used to aid DMD diagnosis and remain an important laboratory test for diagnosis (Van Ruiten et al., 2014). In addition, the exercise protocol used in the present study was able to promote an expressive reduction of muscle strength and the worsening of the inflammatory process and oxidative stress on the diaphragm muscle of *mdx* mice.

The exercised *mdx* mice showed that multiple dystrophic pathological events are targeted by Tempol, which is the focus of our study. Among several antioxidants currently available, Tempol is a redox-cycling, membrane-permeable antioxidant, and is particularly interesting for promoting O_2_ metabolism at rates that are similar to SOD (Batinic-Haberle et al., 2010; Wilcox, 2010). In addition, this antioxidant also facilitates the metabolism of a wide range of ROS and reactive nitrogen species and exhibits catalase activity (Francischetti et al., 2014). Particularly regarding DMD, previous work presented advantageous Tempol effects in the dystrophic skeletal muscle of *mdx* mice, which were partly associated with its anti-inflammatory and antioxidant actions (Hermes et al., 2019, 2020).

In this study, we observed that the Tempol treatment induced an improvement in terms of muscular regeneration, with an increased muscle strength gain and a good correlation with the increase in muscle fiber diameter and CK reduction in the exercise trained-*mdx* mice. Variations in the muscle fiber diameter are normally observed in dystrophic muscles as a result of the different levels of muscle fiber regeneration and recovery of functional innervation (Bulfield et al., 1984). In addition, in agreement with our functional results, previous studies showed that Tempol supplementation increased the dystrophic diaphragm force generation (Burns et al., 2017) and promoted gain in muscle strength in non-exercised *mdx* mice (Hermes et al., 2019).

Regarding the inflammatory process, our data showed that the Tempol treated group presented a decrease in the nuclear factor kappa B (NF-κB) levels. Although the reduction in the NF-κB activation by Tempol has already been shown in another pathology (Cuzzocrea et al., 2001), this is the first study presenting the Tempol action on the NF-κB pathway in the dystrophic muscle. This is a meaningful finding because NF-κB is an important transcription factor that regulates the expression of pro-inflammatory cytokines, such as TNF-α and IL-1β, which were found to have increased in the *mdx* muscle before the period of muscle fiber necrosis (Kumar and Boriek, 2003; Whitehead et al., 2006). Our results showed that the reduction of the NF-κB levels was accompanied by a decrease of the TNF-α content. In agreement with our results, other studies have also demonstrated the effects of Tempol in reducing the inflammatory markers (Gomez-Pinilla et al., 2010; Afjal et al., 2019).

Additionally, the NF-κB levels have also been associated with the vascular endothelial growth factor (VEGF), which is a key regulator of physiological angiogenesis (Neufeld et al., 1999). Kiriakidis et al. (2003) showed that the VEGF production in human macrophages is NF-κB dependent. This close relationship between NF-κB and VEGF may perhaps justify our data regarding this angiogenesis regulator in our experiments. Similar to the NF-κB results, we found that the Tempol treatment also promoted the reduction of the VEGF levels on exercised *mdx* mice, making them closer to the values of the sedentary *mdx* mice. Despite dysfunctional angiogenesis having a significant impact on the dystrophic phenotype and vascular-targeted therapy being proposed as a treatment option for DMD (Podkalicka et al., 2019; Verma et al., 2019), our results suggest that the beneficial effects of tempol observed on the dystrophic muscle are probably not correlated with the angiogenesis pathway.

The NF-κB activation has also been implicated in the amplification of ROS production and vice versa in the dystrophic muscle (Kumar and Boriek, 2003). For instance, in the *mdx* diaphragm muscle, high levels of the oxidative stress markers, such as DHE, lipofuscin granules, and 4-HNE protein adduct, and the NF-κB were shown (Hermes et al., 2016; Mâncio et al.,2017a,b). Similar to a previous study (Mâncio et al., 2017a), we also verified herein that antioxidant treatment promotes the reduction of oxidative stress markers concomitantly with the decrease of the NF-κB levels.

In addition, recently, NF-κB has also been associated in a vicious cycle with peroxisome proliferator-activated receptor-γ coactivator-1α (PGC-1α), where oxidative stress plays an essential role (Rius-Pérez et al., 2020). In our study, we also observed this correlation between the increase of PGC-1α with the concomitant reduction of NF-κB and ROS in exercise-trained-*mdx* mice treated with Tempol. PGC-1α is highlighted as a master regulator of mitochondrial biogenesis and function, including oxidative phosphorylation (OXPHOS) and ROS detoxification (Mastropasqua et al., 2018). A previous study showed that the overexpression of PGC1-α may protect *mdx* muscles by stimulating mitochondrial biogenesis and by providing a calcium sink to limit calcium-related cellular abnormalities, as well as by preventing the activation of cell death pathways associated with mitochondrial permeabilization (Godin et al., 2012). It was also reported that the restoration of the miR-499 expression (which conducts a PGC-1-α dependent mitochondrial oxidative metabolism program) prevented the hallmarks of muscular dystrophy, including the reduction of CK serum release and the improvement of the exercise capacity in *mdx* mice (Liu et al., 2016).

**FIGURE 10:**
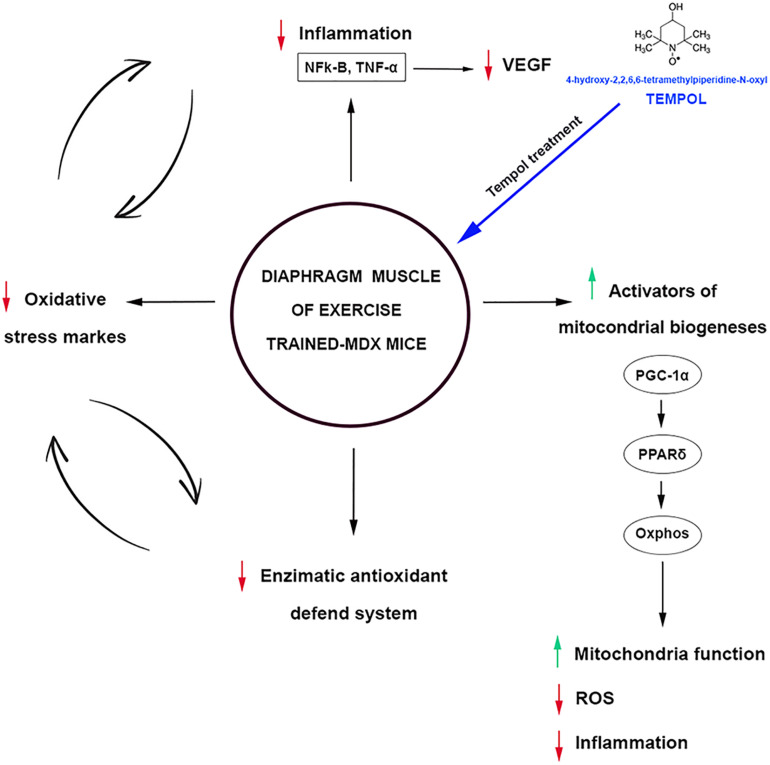
Oxidative stress, inflammation, and activators of mitochondrial biogenesis: targets of Tempol treatment in the diaphragm muscle of exercised trained-*mdx* ↑ mice. ↓ Tempol down-regulated; 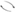 vicious cycle.

Concomitantly with the PGC-1α results, we also found an increase in the Peroxisome proliferator-activated receptor δ (PPARδ) levels in exercise-trained-*mdx* mice treated with Tempol. PGC-1α is reported to be a direct target of PPARδ in the skeletal muscle (Phua et al., 2018). Recently, Bell et al. (2019) showed that PPARδ improves mitochondrial function in the *mdx* mice. We also investigated the mitochondrial respiratory complexes (I, II, III, IV, and V), and similar to the PGC-1α and PPARδ results, the levels of these complexes were up regulated in exercise-trained-*mdx* mice treated with Tempol. Therefore, these results support that modulating PPARδ and PGC-1α can represent an interesting strategy in dystrophy muscular disease, since they are implicated in mitochondria function, oxidative metabolism, ROS detoxification, and regulation of inflammatory cytokines (Figure 10).

Tempol can also act directly in scavenging excessive ROS. It was reported that the increased diaphragm functional capacity in *mdx* mice by Tempol was related to the ability of this antioxidant to scavenge excessive ROS (Burns et al., 2017). Recently, our research group also showed that Tempol improves the redox status in the diaphragm muscle of young *mdx* mice (Hermes et al., 2020). Some studies showed a beneficial Tempol action against oxidative damage in cells by the catalase-like activity, generating H_2_O and O_2_ from H_2_O_2_ and preventing OH production (Wilcox, 2010; Abouzied et al., 2016). This Tempol action may also be applied to our oxidative stress results, justifying the reduction found in oxidative stress markers and enzymatic antioxidant defense system components in the DIA muscle in exercise-trained-*mdx* mice treated with Tempol.

Despite the novelty of our study, some limitations must be recognized. Future studies with wild mice exposed to the same treatment with Tempol and exercise protocol will be useful to understand how changes in the physical activity affect the dystrophic pathology providing further validation for the innovative findings in the current study. In addition, seeing that muscular dystrophy in mice is notoriously variable, a large number of animals should be used when performing experiments with *mdx* mice. Also, although a difference in the inflammatory process morphology was observed between the experimental groups, additional histological methods, such as acid phosphatase, could be useful to complement and strengthen the findings.

To summarize, the striking finding of this work is that the Tempol treated group presented a decrease in the NF-κB in the exercise-trained-*mdx* mice, which is probably related to the ability of this antioxidant to scavenge excessive ROS. Our data also imply that there was an increase in the PGC1-α and PPARδ levels, however, further investigations are required to determine the mechanism by which Tempol modulates these activators of mitochondrial biogenesis. In addition, the present data also reinforce the Tempol as a potential therapeutic for the treatment of DMD.

## Data Availability Statement

The original contributions presented in the study are included in the article/Supplementary Material, further inquiries can be directed to the corresponding author/s.

## Ethics Statement

The animal study was reviewed and approved by Ethics Committee on the Use of Animals (CEUA) of the State University of Campinas (UNICAMP; #4847-1).

## Author Contributions

HS conducted the study. CC, GR, DM, RM, TA, EP, and LK contributed substantially to the acquisition, analysis, and interpretation of data. EM and VC participated in the design of the study and were responsible for the management of grant and coordination. EM, HS, and EP helped in drafting the manuscript. All authors revised it critically for important intellectual content and gave their final approval of the version to be submitted.

## Conflict of Interest

The authors declare that the research was conducted in the absence of any commercial or financial relationships that could be construed as a potential conflict of interest.
