# Supplementary material for: Oxidative Stress, Inflammation, and Activators of Mitochondrial Biogenesis: Tempol Targets in the Diaphragm Muscle of Exercise Trained-mdx Mice
Source: Front Physiol. 2021 Apr 26;12:649793. doi: 10.3389/fphys.2021.649793 (PMC8107395; doi:10.3389/fphys.2021.649793)
Supplement: Supplementary file 1 [file Data_Sheet_1.PDF]

Supplementary Information

OXIDATIVE STRESS, INFLAMMATION AND ACTIVATORS OF MITOCHONDRIAL BIOGENESIS: TEMPOL TARGETS IN THE DIAPHRAGM MUSCLE OF EXERCISE TRAINED-MDX MICE

Heloina Nathalliê Mariano da Silva1, MSc, Caroline Covatti[1], MSc, Guilherme Luiz da Rocha[1], MSc, Daniela Sayuri Mizobuti[1], MSc, Rafael Dias Mâncio[1], PhD, Túlio de Almeida Hermes[1], PhD, Larissa Akemi Kido[1], PhD, Valéria Helena Alves Cagnon[1], PhD, Elaine Cristina Leite Pereira[1,2], PhD, and Elaine Minatel[1]*, PhD

[1] Department of Structural and Functional Biology, Institute of Biology, State University of Campinas (UNICAMP), Campinas, São Paulo 13083-970, Brazil.

[2] University of Brasília (UnB), Faculty of Ceilândia, Brasília, Distrito Federal, 72220-275, Brazil.

Supplementary Figure 1S. Tempol effects on forelimb muscle strength

Representative forelimb muscle strength, assessed by taking measurements of force at time points Start and End, in the 7 animals from each experimental group: (A) sedentary *mdx* mice (*mdx*Sed), (B) exercise trained *mdx* mice (*mdx*Ex) and (C) exercise trained *mdx* mice treated with Tempol (*mdx*Ex+T).

Supplementary Figure 2S. Tempol effects on regenerative muscular process (fibers which central nuclei) and on inflammatory response morphology (inflammatory area)

Representative diaphragm (DIA) cross-sections showing fibers which central nuclei (black arrowheads) and the inflammatory area (outline indicates the representative area) in the 7 animals from each experimental group: sedentary *mdx* mice *(mdx*Sed), exercise trained *mdx* mice (*mdx*Ex) and exercise trained *mdx* mice treated with Tempol (*mdx*Ex+T).

Supplementary Figure 3S. Tempol effects on inflammatory response morphology (macrophage infiltration)

Representative diaphragm (DIA) cross-sections showing the macrophage infiltration (white arrowheads) in the 7 animals from each experimental group: sedentary mdx mice (*mdx*Sed), exercise trained *mdx* mice (*mdx*Ex) and exercise trained mdx mice treated with Tempol (*mdx*Ex+T).

Supplementary Figure 4S. Tempol effects on oxidative stress markers (DHE)

Representative diaphragm (DIA) cross-sections showing the dihydroethidium (DHE) fluorescence in the 7 animals from each experimental group: sedentary *mdx* mice (*mdx*Sed), exercise trained *mdx* mice (*mdx*Ex) and exercise trained *mdx* mice treated with Tempol (*mdx*Ex+T).

Supplementary Figure 5S. Tempol effects on oxidative stress markers (Lipofuscin granules)

Representative diaphragm (DIA) cross-sections showing the autofluorescent lipofuscin granules (white arrowheads) in the 7 animals from each experimental group: sedentary *mdx* mice *(mdx*Sed), exercise trained *mdx* mice (*mdx*Ex) and exercise trained *mdx* mice treated with Tempol *(mdx*Ex+T).

Supplementary Figure 6S. Tempol effects on inflammatory markers (TNF-α and NF-κB)

(A) Representative membranes stained with Ponceau of the different bands shown in this paper. (B) Representative bands analyzed for the tumor necrosis factor-alpha (TNF-α), nuclear factor kappa B (NF-κB) and β-actin quantified by western blotting from the different experimental groups: sedentary *mdx* mice (*mdx*Sed), exercise trained mdx mice (*mdx*Ex) and exercise trained *mdx* mice treated with Tempol (*mdx*Ex+T). The images in Figure 4B represent one animal per group in sequence (n=7 animals per group).

Supplementary Figure 7S. Tempol effects on oxidative stress markers (4-HNE)

(A) Representative membranes stained with Ponceau of the different bands shown in this paper. (B) Representative bands analyzed for the 4-hydroxynonenal (4-HNE)-protein adducts and β-actin quantified by western blotting from the different experimental groups: sedentary *mdx* mice (*mdx*Sed), exercise trained *mdx* mice (*mdx*Ex) and exercise trained *mdx* mice treated with Tempol (*mdx*Ex+T). The images in Figure 7B represent one animal per group in sequence (n=7 animals per group).

Supplementary Figure 8S. Tempol effects on antioxidant system levels (CAT, SOD2, GPx1 and GR)

(A) Representative membranes stained with Ponceau of the different bands shown in this paper. (B) Representative bands analyzed for the catalase (CAT), manganese-superoxide dismutase (SOD2), glutathione peroxidase (GPx1), glutathione reductase (GR) and β-actin quantified by western blotting from the different experimental groups: sedentary *mdx* mice (*mdx*Sed), exercise trained *mdx* mice (*mdx*Ex) and exercise trained *mdx* mice treated with Tempol (*mdx*Ex+T). The images in Figure 8B represent one animal per group in sequence (n=7 animals per group).

Supplementary Figure 9S. Tempol effects on VEGF factor

(A) Representative membranes stained with Ponceau of the different bands shown in this paper. (B) Representative bands analyzed for vascular endothelial growth factor (VEGF) and β-actin quantified by western blotting from the different experimental groups: sedentary *mdx* mice (*mdx*Sed), exercise trained *mdx* mice (*mdx*Ex) and exercise trained *mdx* mice treated with Tempol (*mdx*Ex+T). The images in Figure 9B represent one animal per group in sequence (n=7 animals per group).

Supplementary Figure 10S. Tempol effects on activators of mitochondrial biogenesis and oxidative phosphorylation

(A) Representative membranes stained with Ponceau of the different bands shown in this paper. (B) Representative bands analyzed for peroxisome proliferator-activated receptor δ (PPARδ) and peroxisome proliferator-activated receptor-γ coactivator-1α (PGC-1α) and β-actin quantified by western blotting from the different experimental groups: sedentary *mdx* mice (*mdx*Sed), exercise

trained *mdx* mice (*mdx*Ex) and exercise trained *mdx* mice treated with Tempol (*mdx*Ex+T). The images in Figure 10B represent one animal per group in sequence (n=7 animals per group).

Supplementary Figure 11S. Tempol effects on activators of mitochondrial biogenesis and oxidative phosphorylation

(A) Representative membranes stained with Ponceau of the different bands shown in this paper. (B) Representative bands analyzed for OXPHOS complexes (complex V, III, IV, II and I) and β-actin quantified by western blotting from the different experimental groups: sedentary *mdx* mice (*mdx*Sed), exercise trained *mdx* mice (*mdx*Ex) and exercise trained *mdx* mice treated with Tempol (*mdx*Ex+T). The images in Figure 10B represent one animal per group in sequence (n=7 animals per group).
